# Analysis of COVID-19 under-reporting in Brazil

**DOI:** 10.5935/0103-507X.20200030

**Published:** 2020

**Authors:** Marcelo Freitas do Prado, Bianca Brandão de Paula Antunes, Leonardo dos Santos Lourenço Bastos, Igor Tona Peres, Amanda de Araújo Batista da Silva, Leila Figueiredo Dantas, Fernanda Araújo Baião, Paula Maçaira, Silvio Hamacher, Fernando Augusto Bozza

**Affiliations:** 1 Marketing e Análise, BizCapital - Rio de Janeiro (RJ), Brasil.; 2 Departamento de Engenharia Industrial, Pontifícia Universidade Católica - Rio de Janeiro (RJ), Brasil.; 3 Instituto Nacional de Infectologia Evandro Chagas, Fundação Oswaldo Cruz - Rio de Janeiro (RJ), Brasil.; 4 Instituto D’Or de Pesquisa e Ensino - Rio de Janeiro (RJ), Brasil.

**Keywords:** Covid-19, Coronavirus infections, Reporting of healthcare data, Pandemics/statistics & numerical data, Mortality, Brazil, Covid-19, Infecções por coronavirus, Relatórios de dados de saúde, Pandemias/estatísticas e dados numéricos, Mortalidade, Brasil

## Abstract

**Objective:**

To estimate the reporting rates of coronavirus disease 2019 (COVID-19) cases for Brazil as a whole and states.

**Methods:**

We estimated the actual number of COVID-19 cases using the reported number of deaths in Brazil and each state, and the expected case-fatality ratio from the World Health Organization. Brazil’s expected case-fatality ratio was also adjusted by the population’s age pyramid. Therefore, the notification rate can be defined as the number of confirmed cases (notified by the Ministry of Health) divided by the number of expected cases (estimated from the number of deaths).

**Results:**

The reporting rate for COVID-19 in Brazil was estimated at 9.2% (95%CI 8.8% - 9.5%), with all the states presenting rates below 30%. São Paulo and Rio de Janeiro, the most populated states in Brazil, showed small reporting rates (8.9% and 7.2%, respectively). The highest reporting rate occurred in Roraima (31.7%) and the lowest in Paraiba (3.4%).

**Conclusion:**

The results indicated that the reporting of confirmed cases in Brazil is much lower as compared to other countries we analyzed. Therefore, decision-makers, including the government, fail to know the actual dimension of the pandemic, which may interfere with the determination of control measures.

## Abstract (pt)

**Objetivo:**

Estimar as taxas de notificação de casos de doença pelo coronavírus 2019 (COVID-19) para o Brasil em geral e em todos os estados.

**Métodos:**

Estimamos o número real de casos de COVID-19 utilizando o número de óbitos notificados no Brasil e em cada estado e a proporção entre casos e letalidade, conforme a Organização Mundial da Saúde. A proporção entre casos e letalidade prevista para o Brasil foi também ajustada segundo a pirâmide de idade populacional. Assim, a taxa de notificações pode ser definida como o número de casos confirmados (informados pelo Ministério da Saúde) dividido pelo número de casos previstos (estimado a partir do número de óbitos).

**Resultados:**

A taxa de notificação de COVID-19 no Brasil foi estimada em 9,2% (IC95%: 8,8% - 9,5%), sendo que, em todos os estados, as taxas encontradas foram inferiores a 30%. São Paulo e Rio de Janeiro, os estados mais populosos do país, mostraram baixas taxas de notificação (8,9% e 7,2%, respectivamente). A taxa de notificação mais alta ocorreu em Roraima (31,7%) e a mais baixa na Paraíba (3,4%).

**Conclusão:**

Os resultados indicam que a notificação de casos confirmados no Brasil é muito abaixo da encontrada em outros países que avaliamos. Assim, os responsáveis pela tomada de decisões, inclusive os governos, não têm conhecimento da real dimensão da pandemia, o que pode prejudicar a determinação das medidas de controle.

## INTRODUCTION

The coronavirus disease 2019 (COVID-19) confirmed cases are the most important data to understand the evolution of the disease. However, the rapid spread of the pandemic and the small number of tests performed render it difficult to estimate the actual number of cases and causes under-reporting in different countries. Restricting tests hinder the monitoring of the pandemic progression, resource planning, evaluation of the effectiveness of control measures, as well as benchmarking with other regions and countries. Besides, this could lead to false conclusions that the disease is controlled.

A previous study estimated that only 7.8% of the Brazilian cases are reported.^(1)^ However, this study did not account for variations in COVID-19 mortality by age groups, which is considered relevant by the World Health Organization (WHO).^(2)^ Also, the above-mentioned study presented the rates at a national level, not accounting for differences among the regions of each country, and this is particularly important in Brazil due to its continent-sized area.

Therefore, this study aimed to estimate the under-reporting of COVID-19 cases in Brazil. We took into consideration the variations in mortality by age group and the confirmation-to-outcome delay to estimate the actual proportion of reported cases. These rates may evidence the frailty of the official numbers and assist decision-makers in the management of new policies and measures to control the pandemic.

## METHODS

We performed a cross-sectional study to estimate the under-reporting rates of COVID-19 across Brazil as a whole and by state, using both national and international data. We included secondary data about the number of confirmed cases and deaths in Brazil reported by the Ministry of Health,^(3)^ the number of cases and deaths worldwide provided by the European Centre for Disease Prevention and Control,^(4)^ Brazil’s age pyramid provided by the Brazilian Institute of Geography and Statistics (*Instituto Brasileiro de Geografia e Estatística* - IBGE),^(5)^ and the age pyramid of other countries provided by the United Nations.^(6)^

Our main outcome was the reporting rate, i.e., the percentage of reported COVID-19 confirmed cases. This was calculated as the proportion between the base case-fatality ratio (base CFR) and the observed case-fatality ratio (observed CFR.)^(1)^ The base CFR is defined as the number of deaths over the number of cases of the disease.^(7)^ We used the CFR estimates from the observed COVID-19 deaths and confirmed cases in China as our best base CFR estimate.^(1,8)^ Also, a study performed by the WHO^(2)^ suggested that the probability of death due to COVID-19 varies substantially according to the patient’s age. Thus, we considered the observed CFR as the age-adjusted CFR from Brazil, using the mortality of the WHO study stratified by age groups.

### Statistical analysis

The observed CFR was adjusted to the confirmation-to-outcome (recovery or death) delay using a lognormal distribution with an average of 13 days and a 12.7 days standard deviation (SD).^(9)^ Next, we calculated the reporting rate of COVID-19 cases for Brazil and each Brazilian state. We evaluated the 95% confidence intervals (95%CI) using a binomial test, taking the number of deaths as “successes” and the number of outcomes as the total sample size. The higher the difference between the observed and the base CFR, the lower the reporting rate.

The reporting rates of other countries (in particular from South Korea, Germany, the United States, Italy, and Spain) were also estimated for comparison with the Brazilian case. One base CFR was used for all these countries, calculated as their average considering age pyramids.

## RESULTS

As of April 20, 2020, Brazil had 40,581 reported COVID-19 cases and 2,575 deaths, representing a gross CFR of 6.3% (Table 1). Using the confirmation-to-outcome delay, we estimated 18,150 cases with an outcome, with an observed CRF of 14.2%. Because Brazil had a younger population as compared to other countries, the base (age-adjusted) CFR was 1.3%. Using our method, we concluded that the estimated reporting rate of confirmed COVID-19 cases in Brazil was around 9.2% (95%CI 8.8% - 9.5%). Therefore, the actual number of cases in Brazil was about 11 times higher than the officially reported cases.

**Table 1:** Number of cases, deaths, and reporting rates in Brazil (as of April 20)

Brazil	
Reported cases	40,581
Reported deaths	2,575
Cases with outcome	18,150
Crude CFR	6.3
Observed CFR	14.2
Base CFR	1.3
Reporting rate (95%CI)	9.2 (8.8% - 9.5%)

CFR - case-fatality ratio. Results expressed as n or %.

**Figure 1:**
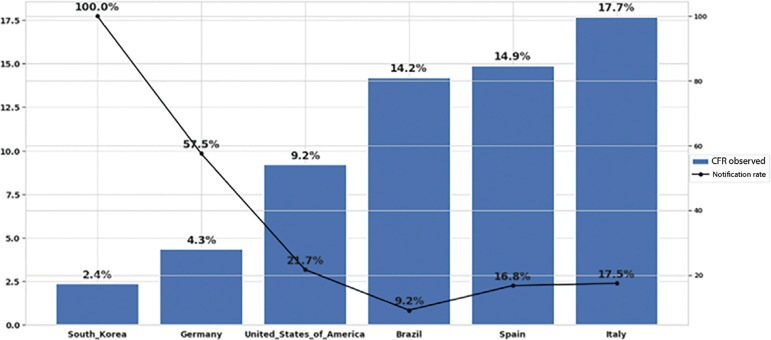
Reporting rate and the observed case-fatality ratio in selected countries. CFR - case-fatality ratio.

Comparing the observed CFR and the estimated reporting rates between Brazil and other countries (Figure 1), one can note that Spain and Italy showed the highest observed CFR rates and the lowest reporting rates. Conversely, the United States, Germany, and South Korea had lower CFR but higher reporting rates.

**Figure 2:**
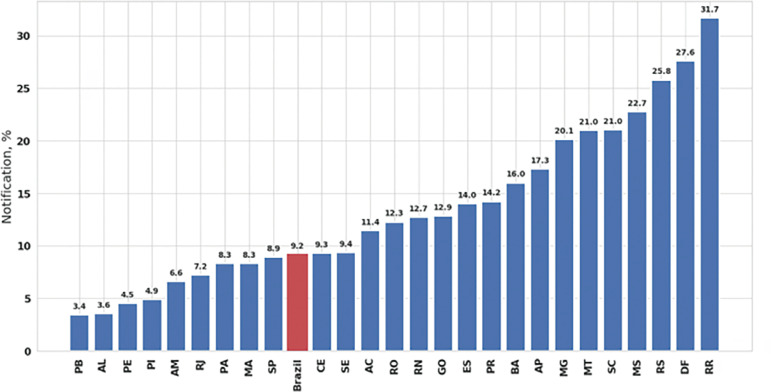
Reporting rates in Brazilian states.

There was high variability among the estimated reporting rates in Brazilian states (Figure 2 and Table 2). Out of its 27 states, 17 (63%) presented a reporting rate higher than the overall reporting rate of Brazil. Also, all the states showed reporting rates below 32%. São Paulo and Rio de Janeiro, the most populated states in Brazil, showed low COVID-19 reporting rates (8.9%; 95%CI 8.7% - 9.1% and 7.2%; 95%CI: 7.1% - 7.3%, respectively). States with fewer deaths until April 20 had higher confidence intervals. Roraima had the highest reporting rate (31.7%; 95%CI: 12.2% - 106.3%]; however, it also presented the lowest number of reported deaths, resulting in a high variability estimate (Table 2).

**Table 2:** Number of cases, deaths, and reporting rates in Brazilian states (as of April 20)

State	Reported cases	Reported deaths	Reporting rate (95%CI)
Paraíba	245	32	3.4 (2.9 - 4.3)
Alagoas	171	18	3.6 (2.7 - 4.9)
Pernambuco	2,690	234	4.5 (4.4 - 4.7)
Piauí	158	12	4.9 (3.4 - 7.9)
Amazonas	2,160	185	6.6 (6.3 - 7)
Rio de Janeiro	4,899	422	7.2 (7.1 - 7.3)
Pará	902	35	8.3 (6.7 - 10.6)
Maranhão	1,320	54	8.3 (7 - 9.9)
São Paulo	14,580	1,037	8.9 (9.1 - 8.7)
Ceará	3,482	198	9.3 (8.8 - 9.8)
Sergipe	86	5	9.4 (4.8 - 21.3)
Acre	176	8	11.4 (6.6 - 22.4)
Rondônia	160	4	12.3 (5.5 - 31.8)
Rio Grande do Norte	595	27	12.7 (9.6 - 17.3)
Goiás	403	19	12.9 (9.1 - 18.7)
Espírito Santo	1,168	33	14 (11 - 18.3)
Paraná	1,007	51	14.2 (11.8 - 17.2)
Bahia	1,341	46	16 (13.1 - 19.8)
Amapá	433	13	17.3 (11.3 - 28.5)
Minas Gerais	1,189	41	20.1 (16.2 - 25.2)
Mato Grosso	181	6	21 (10.9 - 43.5)
Santa Catarina	1,025	35	21 (16.5 - 27.3)
Mato Grosso do Sul	171	5	22.7 (10.7 - 52.4)
Rio Grande do Sul	889	27	25.8 (19.3 - 35)
Distrito Federal	872	24	27.6 (20.2 - 38.6)
Roraima	244	3	31.7 (12.2 - 106.3)

Results expressed as n or %.

## DISCUSSION

The number of COVID-19 confirmed cases in Brazil has been highly under-reported. In this work, we estimated that the actual number of cases has been about 11 times higher than the currently reported. Also, there was high variability in the reporting rate of COVID-19 cases among Brazilian states. As of April 20, São Paulo and Rio de Janeiro together account for 48% of the total reported cases in the country and presented rates that are lower than those from other Brazilian states.

The under-reporting rate noticed in Brazil may be related to some factors, such as operational difficulties to test the population leading to an extended delay between tests and results, the lack of new tests, and the guidance to test only more severe cases.^(10,11)^ Also, the capacity for obtaining test results is variable among hospitals and institutions. While tests to be confirmed pile up, the number of reported deaths is also delayed.^(12)^ Hence, under-reporting has not yet a trend to decrease, as shows the temporal variation analysis by Russell et al.^(1)^ (temporal plot of Brazil accessed on April 21).

The reporting rates are different among the Brazilian states. The difference between the highest and lowest reporting rates (31.7% in Roraima - North region and 3.4% in Paraiba - Northeast region) suggests distinct testing and reporting confirmed cases policies. We emphasize that states with a lower number of deaths or cases have higher uncertainty on their reporting rates. However, we observed that all states present high under-reporting levels, which represents a concern for decision-makers, as it could mislead the analysis of the disease control and control measures.

Our methodology was based on the CFR under-reporting estimation proposed by Russell et al.,^(1)^ however we added an age-adjustment for the observed CRF. Since Brazil has a younger population as compared to Italy and Spain, the expected CFR tends to be lower (a CFR of 1.3% estimated for Brazil and 2.5% for both). Therefore, the estimated reporting rate is also lower in Brazil. Thus, age-adjustment provides precise estimation for the base CFR, as age is one of the risk factors for severe COVID-19.^(13)^

This study has limitations. First, to calculate the reporting rate we based our COVID-19 base CFR mainly on Chinese data. Second, we took into consideration a delay curve between notification and outcome to calculate the actual (observed) CFR as a lognormal distribution as an average of 13 days (SD: 12.7 days).^(9)^ However, the current status of the disease may provide different estimates for the confirmation-to-death delay and thus, any variation may influence the final results. Third, the age-adjustment of other countries CRF was based on a set of reference countries (South Korea, Germany, the United States, Spain, and Italy) and their age groups. Then, as distribution of COVID-19 confirmed cases may vary according to the region, this could also impact the results. Fourth, the lack of standardization in the reporting of death due to the disease can provide inaccurate CFR estimates, as different countries have adopted varied policies cause of death reporting. Moreover, Brazilian states show different disease progression curves, which may also bias the results, especially for states with fewer reports.

## CONCLUSION

This study assessed the underreporting of COVID-19 confirmed cases in Brazil and its states. The results indicate that the reporting of confirmed cases in Brazil represented only 9.2% (CI95% 8.8% - 9.5%) of the actual figures, much lower than the observed in the other countries. Therefore, decision-makers and governments cannot rely on their reporting sources and have to provide measures to control a pandemic, which real dimension they do not know.
